# Coexistence of Ehlers-Danlos syndrome and multiple sclerosis

**Published:** 2015-04-04

**Authors:** Hatice Kose Ozlece, Faik Ilik, Nergiz Huseyinoglu

**Affiliations:** 1 Department of Neurology, School of Medicine, Kafkas University, Kars, Turkey; 2 Department of Neurology, School of Medicine, Mevlana University, Konya, Turkey

**Keywords:** Ehlers-Danlos Syndrome, Multiple Sclerosis, Coexistence, Neurological Manifestations

A 26-year-old male patient was admitted to our outpatient clinic with complaints of numbness and weakness in his left arm and leg. These complaints had started about 10 days ago and were continuing. From his personal history, it was found out that he had been followed up with the diagnosis of Ehlers-Danlos syndrome (EDS) hypermobile tip for 10 years, and that he had had low vision in his left eye lasting for 15 days, but he had not consulted a physician for this complaint. After 1 year had passed from the complaint of blurred vision, he had had a weakness in his right arm and loss of balance, but again he had not sought medical advice. From his familial history, it was found out that his paternal grandmother, father, aunt, cousins, and younger sister were being followed up with the diagnosis of EDS. General physical examination revealed no pathological features except for several atrophic cicatrices on the face, back, and arms and hyperflexibility of the joints (Figure 1). No cardiologic or ophthalmologic involvement was detected in terms of EDS. Neurological examination revealed that the patient is conscious, cooperated and oriented. His cranial nerve examinations were normal. His muscle strength was 3/5 in the upper left limb, 4/5 in the lower left limb, and 5/5 in the upper and lower right limbs. Left-sided hemihypoesthesia including the face, and globally hyperactive deep tendon reflexes were seen. The patient had bilateral extensor plantar reflexes and reduced abdominal cutaneous reflex on the left side. His cerebellar tests were normal, and no urinary or fecal incontinence. His cranial magnetic resonance imaging (MRI) revealed several ovoid-shaped periventricular lesions located perpendicular to the ventricle, which is consistent with demyelinating plaques (Figure 2). His cervical MRI revealed several centrally-located, ovoid-shaped lesions aligned with C2-C3-C4 segments, which are consistent with demyelinating plaques. His thoracic MRI was considered to be normal. For the patient, who had blurred vision, a visual evoked potential (VEP) test was performed. p100 wave latency was lengthened for both VEP systems (right: 136, left: 132). Brainstem auditory evoked potential examination was normal. ANA, anti-dsDNA, Anti-cardiolipin IgG and IM, Homocysteine, Lupus Anticoagulant, Protein C, Protein S, Prothrombin II gene mutation, Factor 5 leiden mutation investigations revealed no significant pathologies. The patient was evaluated according to McDonald’s criteria, an acute multiple sclerosis (MS) episode was considered, and pulse steroid therapy was prescribed for 10 days. Following the treatment, his symptoms at admission were recovered. He is now being followed up by our outpatient clinic.

EDS is a rare, inherited disease characterized by disturbed collagen synthesis and enzyme dysfunction. Central nervous system involvements are remarkable, mainly in vascular type EDS.^1^ However, only one study could be identified that examined the potential association with MS, and it was emphasized in this study that MS prevalence is 10 to 11-fold greater in EDS patients compared to general population.^2^

**Figure 1:**
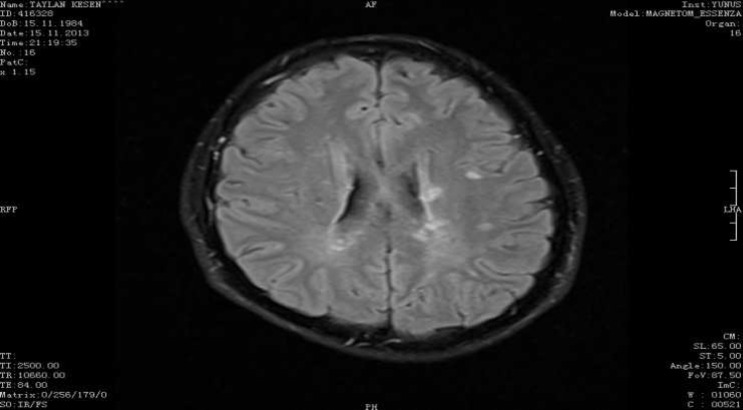
General physical examination revealed hyperflexibility of the joints

**Figure 2:**
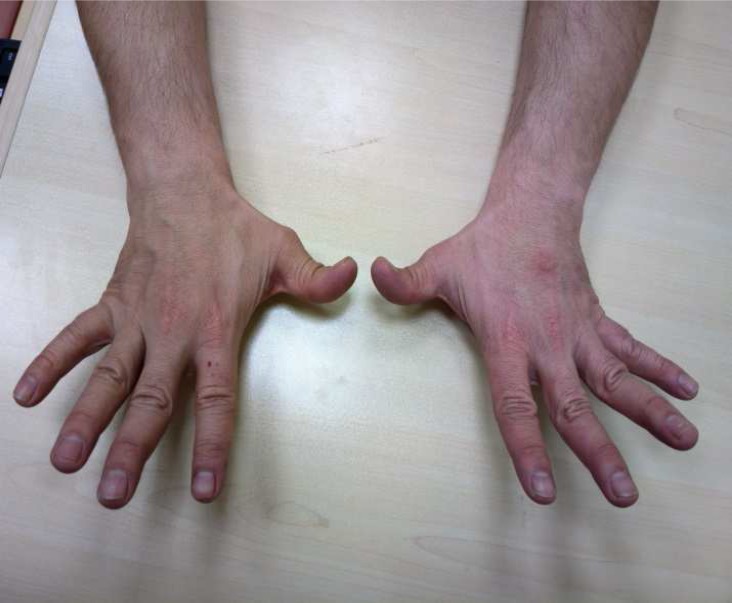
Hyperintense, ovoid-shaped, periventricular lesions located perpendicular to the ventricle

ECM is a structure that encloses and supports the cells. It has three major components. Structural proteins (mainly collagen), proteoglycan and hyaluronan, and specialized multi-adhesive proteins. ECM proteins were observed to involve in central nervous system inflammation and demyelination.^3^ It is thought that in EDS, there are mutations in the genes coding ECM proteins. These are cells which ensure cell migration and organization, and EDS is seen when a synthesis defect occurs in their synthesis.^4^^,^^5^ ECM proteins are produced by oligodendrocytes and astrocytes in the central nervous system, and were observed to be associated with the astroglial response in the MS lesions. According to a hypothesis, it can show effects on connective tissues and at the vascular level. Changes in the ECM proteins such as collagen and tenascin which are present in the blood vessel walls can cause myelin destruction by increasing the migration of the immune cells to the central nervous system. According to another hypothesis, MS-EDS association may be due to a suspicious gene. There may be a polygenic effect in MS, and one of these mutations may cause EDS. There are some points to be considered in the treatment of MS and EDS. These patients, who undergo physiotherapy due to MS, strong passive exercises can trigger pain or cause joint dislocations. Neck extension should be avoided to prevent carotid artery dissection.^2^

In our case, we discuss a rare association in terms of their underlying mechanisms and of the points to be considered in the treatment.

## Notes:

**How to cite this article: **Ozlece HK, Ilik F, Huseyinoglu N. Coexistence of Ehlers-Danlos syndrome and multiple sclerosis. Iran J Neurol 2015; 14(2): 116-7.
